# Burnout among trauma surgeons: a systematic review and meta-analysis

**DOI:** 10.1136/tsaco-2025-001873

**Published:** 2025-10-28

**Authors:** Sebastian Kirdar-Smith, Alec Knight, Ricardo Twumasi

**Affiliations:** 1 King’s College London, London, England, UK

**Keywords:** burnout, meta-analysis, Education, Medical

## Abstract

**Background:**

Burnout is increasingly recognized as a critical occupational issue impacting physician well-being and patient care. Although surgeons are known to experience high burnout rates, the specific burden among trauma surgeons remains poorly researched. This systematic review and meta-analysis focuses on burnout exclusively among trauma surgeons. We aim to analyze the prevalence of burnout among trauma surgeons and identify associated factors by analyzing their alleviating and exacerbating influences through systematic review, meta-analysis, and meta-regression.

**Methods:**

Following PRISMA (Preferred Reporting Items for Systematic Reviews and Meta-Analyses) and MOOSE (Meta-analysis of Observational Studies in Epidemiology) guidelines, we used a combination of searching databases, individual journals and cross-referencing. Two independent reviewers screened studies measuring burnout in trauma surgeons. A random-effects meta-analysis was performed using logit-transformed proportions. Heterogeneity was assessed using I² statistics and meta-regression examined the impact of measurement tools.

**Results:**

Analysis of 19 studies (n=4,634) revealed a pooled burnout prevalence of 60.0% (95% CI 46.9% to 74.4%) with substantial heterogeneity (I²=97.9%, p<0.0001). Studies using the Maslach Burnout Inventory (n=13) showed high emotional exhaustion (35.2%) and depersonalization (45.6%), but maintained strong personal accomplishment (75.3%). Key burnout-exacerbating factors included younger age, long working hours, and administrative burden, whereas protective factors included mentorship and protected non-clinical time.

**Conclusions:**

Trauma surgeons experience among the highest burnout rates reported among surgical specialties, warranting systemic physician-centric interventions, with a shift in focus from diagnosis to prevention. Despite significant occupational stressors, persistently high personal accomplishment levels suggest specialty-specific resilience factors, meriting further investigation. Evidence-based strategies, including formal mentorship programs, psychological risk management models, and protected non-clinical time have the potential to mitigate burnout.

**WHAT IS ALREADY KNOWN ON THIS TOPIC**

- The phenomenon of burnout is prominent within the field of medicine, and especially so within surgery. It is commonly recognized that burnout and stress disorders in trauma surgeons are high, resulting in some of the lowest mental quality of life and highest incidence of burnout among all specialties.

**WHAT THIS STUDY ADDS**

- Although there are studies including trauma surgeons in part, this is the first review and meta-analysis to focus on burnout syndrome exclusively among trauma surgeons. This systematic review and meta-analysis of 19 studies comprising 4634 trauma surgeons found a pooled burnout prevalence of 60.0% (95% CI 46.9% to 74.4%), significantly higher than other surgical specialties.

**HOW THIS STUDY MIGHT AFFECT RESEARCH, PRACTICE OR POLICY**

- These findings reinforce the necessity to intervene in an evidence-based manner, putting the surgeon first, and accepting, on an organizational level, that burnout is an occupational phenomenon.

## Introduction

 Burnout is a syndrome resulting from unsuccessfully managed chronic workplace stress. This is most commonly denoted by the presentations of exhaustion, cynicism, and diminished productivity^1^.

Burnout is a relatively new concept, first theorized in the 1970s, with most research conducted in the last 20 years. Nevertheless, burnout is well researched and has been the focus of many studies throughout recent decades.^2^ However, few systematic reviews focusing on physician burnout by specialty exist, and none aim exclusively at burnout in trauma surgeons.

Since burnout was first hypothesized, various metrics for measuring burnout have been developed and are in current use. The most widely used tool is the Maslach Burnout Inventory (MBI),^3^ which psychometrically analyzes three distinct areas of burnout: emotional exhaustion (EE), depersonalization (DP), and reduced personal accomplishment (PA). However, limitations exist: the MBI conceptualizes burnout as a spectrum rather than a dichotomous trait, there are no universally accepted cut-off scores, and accordingly, the tool is not clinically diagnostic of burnout.

The proximal effects of burnout on physicians are often crucially overlooked and left unaddressed by governing bodies. Both the individual and organizational ramifications of burnout are severe, and if left unaddressed, can have profound impacts on the quantity and quality of patient care.^4^ Moreover, burnout is strongly associated with actual reductions in professional work effort,^5^ resulting in medical errors, higher healthcare costs, lower quality of care, and worse patient outcomes.^6^

The phenomenon of burnout is especially prominent within the field of surgery, as surgeons experience the highest rates of burnout among doctors^7^. Burnout is especially prevalent in the surgical specialty of trauma. It is commonly recognized that burnout and stress disorders in trauma surgeons are high,^8^ resulting in some of the lowest mental quality of life and highest incidence of burnout among all specialties.^9^ Stress is ubiquitous in the field of medicine, with trauma surgeons in particular experiencing frequent periods of high stress and volatile conditions,^10^ leading to a higher susceptibility for developing burnout. This aligns with existing research hypothesizing that burnout is more likely to occur among trauma surgeons.^4^

The objective of this systematic review and meta-analysis was to examine burnout prevalence among trauma surgeons. Thereafter, we aimed to identify associated factors and assess alleviating or exacerbating effects on rates of burnout.

## Materials and methods

**Figure 1:**
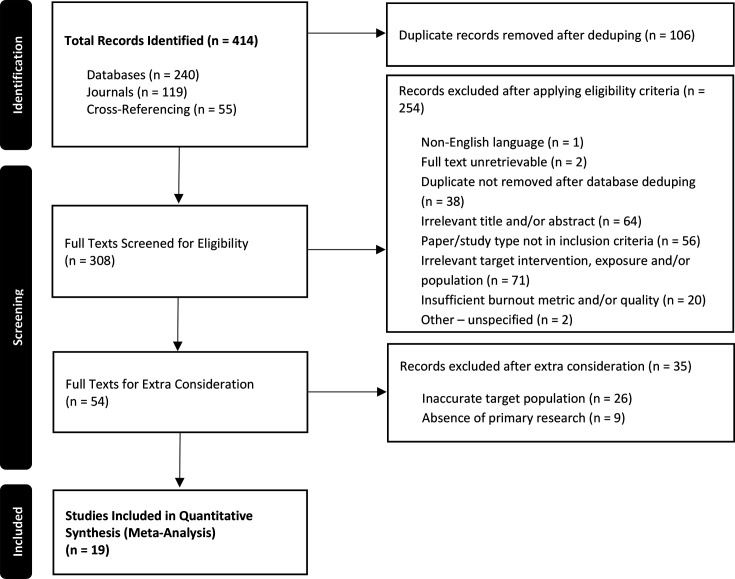
PRISMA flowchart. PRISMA, Preferred Reporting Items for Systematic Reviews and Meta-Analyses.

We performed a systematic review and meta-analysis to analyze burnout exclusively among trauma surgeons. This study was conducted adhering to an established and accepted protocol (CRD42024586591),^11^ and follows the Preferred Reporting Items for Systematic Reviews and Meta-Analyses (PRISMA) (figure 1) and MOOSE (Meta-analysis of Observational Studies in Epidemiology) (online supplemental figure 1) protocols.

Per our protocol,^11^ two review teams were involved throughout the methods process. The primary reviewer (SK-S) performed all search strategies, applied eligibility criteria, and performed quality assessment, data extraction, and statistical analyses. A second review team independently assessed the decisions of the primary reviewer, which ensured both an error-mitigation system and reducing any unintentional bias. In the case of conflicting views, a two-stage discrepancy resolution and arbitration system was implemented, as outlined in the protocol.^11^

### Data sources and search strategy

The literature search process consisted of three stages: searching databases, hand-searching journals, and cross-referencing. The following databases were searched through the Ovid platform identifying 240 records: MEDLINE/PubMed, EMBASE, and PsycINFO. Hand-searching journals yielded a further 119 records, with the remaining records (n=55) originating from cross-referencing. All platform, database, and journal searches were conducted during the same date range (inception through July 30, 2024).

For searches, we used combinations of: MeSH terms, title words, text words, and keywords. There was a predominant focus on identifying observational (mainly cross-sectional) studies. Full texts of all non-duplicate records (n=308) were screened to ensure all appropriate data were assessed for eligibility.

### Study eligibility criteria

Articles retrieved from all search modalities after automatic deduplication, were screened independently by two research teams—first by title/abstract, and again by full text review. The inclusion criteria involved a stepwise strategy for article screening, requiring the need for: (1) generating primary research; (2) specific subject targeting, in full or in part, of trauma surgeons; (3) utilization of a validated burnout metric; and (4) production of statistics for burnout prevalence, or if not, sufficiently detailed data to calculate one. After the application of the study eligibility criteria, 54 records remained. A third round of in-depth screening and review was conducted, leaving 19 full-text articles meeting inclusion criteria for both quantitative and qualitative synthesis.

### Quality assessment and risk of bias

Quality assessment and risk of bias were evaluated by application of the Newcastle-Ottawa Scale (NOS) adjusted for cross-sectional studies. The NOS tool assessed studies according to the academic caliber of three study dimensions: selection, comparability, and outcome. This allowed us to formulate a quality assessment and risk of bias score out of 9, enabling analysis of areas of methodological quality that are poorly addressed in the included studies.^12^ Therefore, this also informed the level of certainty of evidence in our included studies. Additionally, missing information deemed sufficiently important to the integrity of the study is reported as such, so as to minimize reporting bias.

### Outcome measures

The primary outcome measure was prevalence of burnout. Burnout prevalence can be measured by numerous tools, the most common of which is the MBI. There is an academic predominance of the MBI, with one study finding that 85.7% of their 182 included studies used a version of the MBI.^3^ The relative homogeneity of burnout metrics used in literature is academically advantageous as it allows for inter-study comparison and collation of data for meta-analyses. Secondary outcome measures primarily included factors that alleviate or exacerbate burnout.

### Data extraction and synthesis

To ensure organizational integrity in processing and cataloging screened studies, each record was assigned an internal identifier number before initiating any data extraction. Employing a standardized form, we extracted burnout prevalence statistics, along with accompanying relevant demographic and statistical data. Reported demographic data predominantly included age, sex, and family status. Other variables, notably race, were not commonly reported, thus we were not able to confidently investigate such variables. Data extraction was completed by SK-S, with an additional review team (RT and AK) checking every record. Where confidence intervals or variance were missing, authors were contacted; 3 of 19 responded. Where authors provided sufficient primary data, these data were used directly in our analysis. Where we did not receive sufficient data, these data were instead computed using the escalc feature of metafor, which uses logit distribution to estimate confidence from the point estimate and sample size.

### Quantitative analysis

Meta-analyses were performed using R V.4.4.1 and the metafor package. Effect sizes were calculated using logit-transformed proportions to adjust the data distribution to meet the statistical assumptions of normality. Logit proportions were back transformed to raw proportions for ease of clinical interpretation. Inverse variance weighting was used. A random-effects model was fitted using restricted maximum likelihood estimation with Knapp and Hartung adjustment for test statistics. Heterogeneity was assessed using I² statistics and Cochran’s Q test. Publication bias was evaluated using funnel plot visualization, Egger’s test, and Duval and Tweedie’s trim-and-fill method. A cumulative meta-analysis was conducted to examine the evolution of evidence over time. Sensitivity analysis was completed through a cumulative meta-analysis, assessing the change in model over time as each study was added. A subgroup analysis compared studies using the MBI with those using other measurement tools.

To examine the impact of measurement tools, we conducted a mixed-effects meta-regression with MBI usage as a moderator variable. The model included assessment of residual heterogeneity, moderator testing using F-statistics, and calculation of the proportion of heterogeneity explained (R²). Predicted values and confidence intervals were back-transformed to the proportion scale for interpretation.

## Results

The PRISMA flowchart is shown in figure 1. A total of 414 records were identified in the literature search. After applying the eligibility criteria to the full texts of all deduplicated records, 308 remained. This left 54 studies for extra consideration by both reviewer teams, enacting a more thorough screening process, as per our published protocol^11^. At the end of the selection process, a total of 19 studies met the inclusion criteria in full, and thus qualified for data extraction.

### Study characteristics

The 19 included studies (table 1) involved 4634 trauma surgeons, with an average sample size of 244 (range: n=8–1419). 15 studies in the review were published within the last 5 years, demonstrating the recency of primary evidence-producing articles on burnout. 13 studies used either the MBI or validated variations of it, the remainder (n=6) used validated auxiliary tools. Although there are no universally recognized cut-off scores for the NOS, based on published statistical reliability tests, we categorized results into three classes: high risk (0–3 NOS score), moderate risk (4–6), and low risk of bias (7–9).^13^ Given this evidence-based approach, our review contained 73.7% (n=14) “low risk of bias” studies,^914^,^26^ 26.3% (n=5) “moderate risk of bias” studies,^27^,^31^ and no high risk of bias studies.

**Table 1:** Table of included studies and selected characteristics

Study (n=19)	Country	Study participants (n)	Trauma surgeons in sample (n)	Burnout metric	Burnout rate*	NOS QA and RoB (/9)
Sanchez-Madrid *et al*^18^	Spain	149	149	MBI	83.2%^(c)^	7
Balch *et al*^17^	USA	7,799	341	MBI	45.3%^(c)^	7
Balch *et al*^9^	USA	7,192	345	MBI	51.6%	8
Faivre *et al*^22^	France	107	107	MBI	40.2%	7
Faivre *et al*^21^	France	441	441	MBI	38.9%	8
Caesar *et al*^16^	UK	165	27	CBI	40.2%	8
Driesman *et al*^20^	USA	27	27	MBI	64.6%^(c)^	7
Elkbuli *et al*^28^	USA	276	276	Survey	93.8%	6
Maharjan *et al*^27^	Nepal	34	34	MBI	97.1%^(c)^	5
Vaisman *et al*^31^	Chile	136	136	MCQs	53.7%	5
Brown *et al*^30^	USA	291	291	Survey	61.0%	5
Houdmont *et al*^14^	UK	571	55	MBI-HSS MP	56.4%^(c)^	7
Huang *et al*^19^	USA	7,375	1,419	aMBI-HSS MP	47.5%^(c)^	9
Nugent *et al*^23^	USA	149	8	aMBI-HSS	62.5%	7
Coleman *et al*^15^	USA	181	181	MBI	40.4%	8
Hamdan *et al*^24^	Jordan	135	29	aMBI	45.2%	8
Olate *et al*^26^	Chile	99	99	MBI-HSS MP	35.0%	7
Davis *et al*^29^	USA	159	159	Survey	41.0%	5
Nayar *et al*^25^	UK	369	369	OLBI	90.4%	7

* No specific burnout statistic; prevalence has been calculated from available data in the article—marked appropriately with a superscript (c), (eg, x.xx%(c)).

CBI, Copenhagen Burnout Inventory; MBI, Maslach Burnout Inventory; MBI-HSS, Maslach Burnout Inventory - Human Services Survey; MBI-MP, Maslach Burnout Inventory for Medical Personnel; MCQs, multiple choice questions; NOS, Newcastle-Ottawa Scale; OLBI, Oldenburg Burnout Inventory; QA, quality assessment; RoB, risk of bias.

### Prevalence of burnout in trauma surgeons

**Figure 2:**
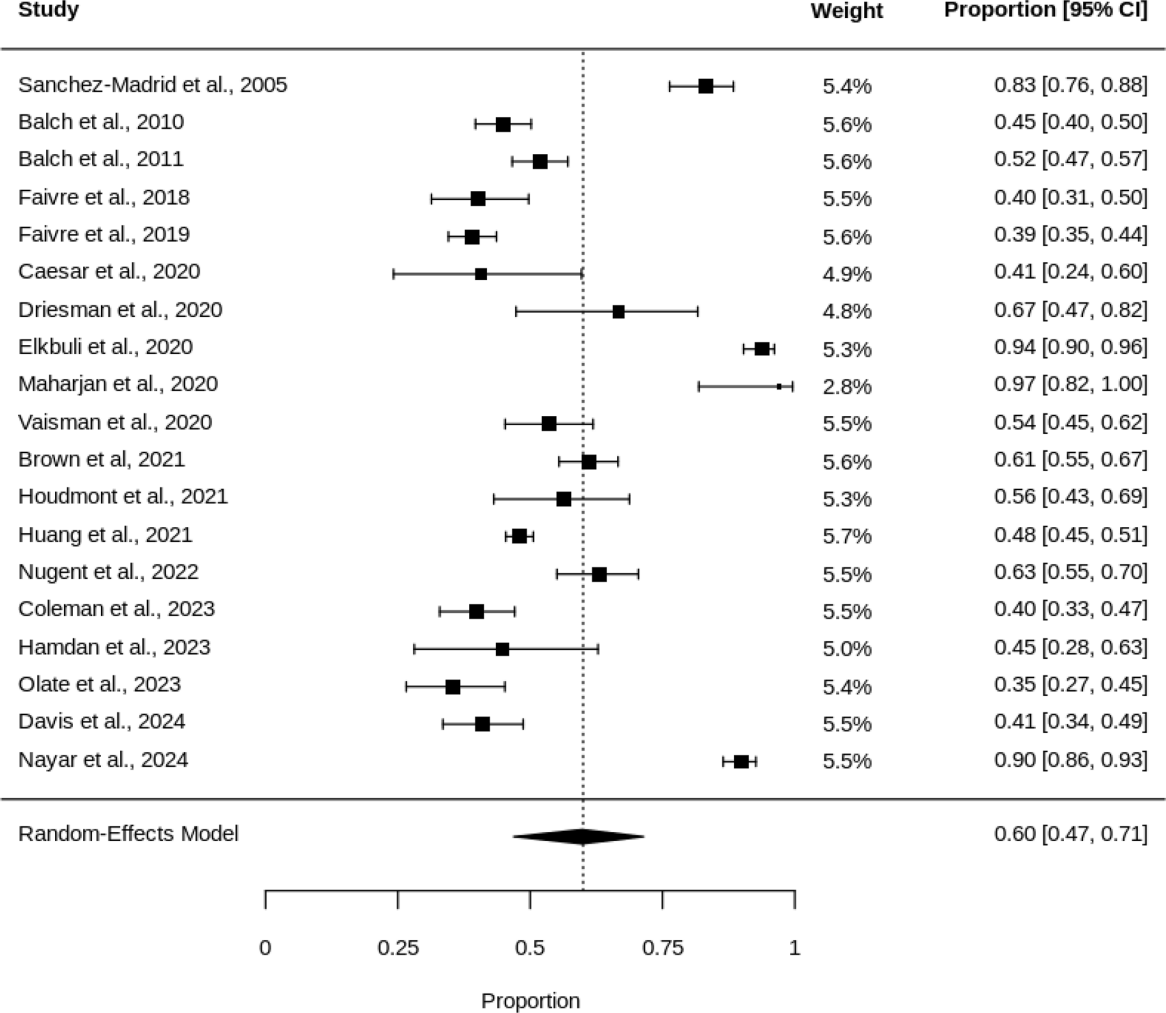
Forest plot of burnout prevalence in trauma surgeons.

The pooled prevalence of burnout was 60.0% (95% CI 46.9% to 74.4%) per figure 2. Substantial heterogeneity was observed (I² = 97.9%, Q=421.31, p<0.0001), indicating significant variation in burnout prevalence across studies.

The prevalence of burnout among trauma surgeons ranged from 38.9%^21^ to 97.1%^27^. Nine of the included studies provided complete information regarding all three subcomponents of the MBI. Therefore, these were tabulated together in table 2, and used to calculate a sample size-weighted average for each individual subcomponent.

**Table 2:** Table of studies using the MBI or MBI subvariants

Study (n=9)	Trauma surgeon sample size (n)	High EE	MBI High DP	Low PA
Sanchez-Madrid *et al*^18^	8	44.1%	64.6%	24.5%
Faivre *et al*^22^	107	26.2%	63.6%	33.6%
Faivre *et al*^21^	441	13.8%	22.7%	18.6%
Driesman *et al*^20^	27	11.1%	44.4%	14.8%
Maharjan *et al*^27^	34	94.1%	100.0%	29.4%
Houdmont *et al*^14^	55	60.0%	52.7%	12.7%
Nugent *et al*^23^	149	62.5%	62.5%	12.5%
Hamdan *et al*^24^	29	44.4%	13.3%	0.7%
Olate *et al*^26^	99	55.0%	61.0%	76.0%
	**Weighted averages**	**35.2%**	**45.6%**	**24.7%**

DP, depersonalization; EE, emotional exhaustion; MBI, Maslach Burnout Inventory; PA, personal accomplishment.

On the EE component of the MBI, an average of 35.2% (range: 11.1%^20^–94.1%^27^) of trauma surgeons showed high EE scores. On the DP component of the MBI, an average of 45.6% (range: 22.7%^21^–100.0%^27^) showed high DP scores. However, on the reduced feelings of PA component of the MBI, a relatively low 24.7% (range: 0.7%^24^–33.6%^22^) showed low PA scores. Our findings of high EE and DP averages substantiate claims that trauma surgery is an intensely stressful and exhausting specialty, and the PA average validates the idea that trauma surgery is highly rewarding, as relative PA levels are persistently high.

### Statistical analysis

**Figure 3:**
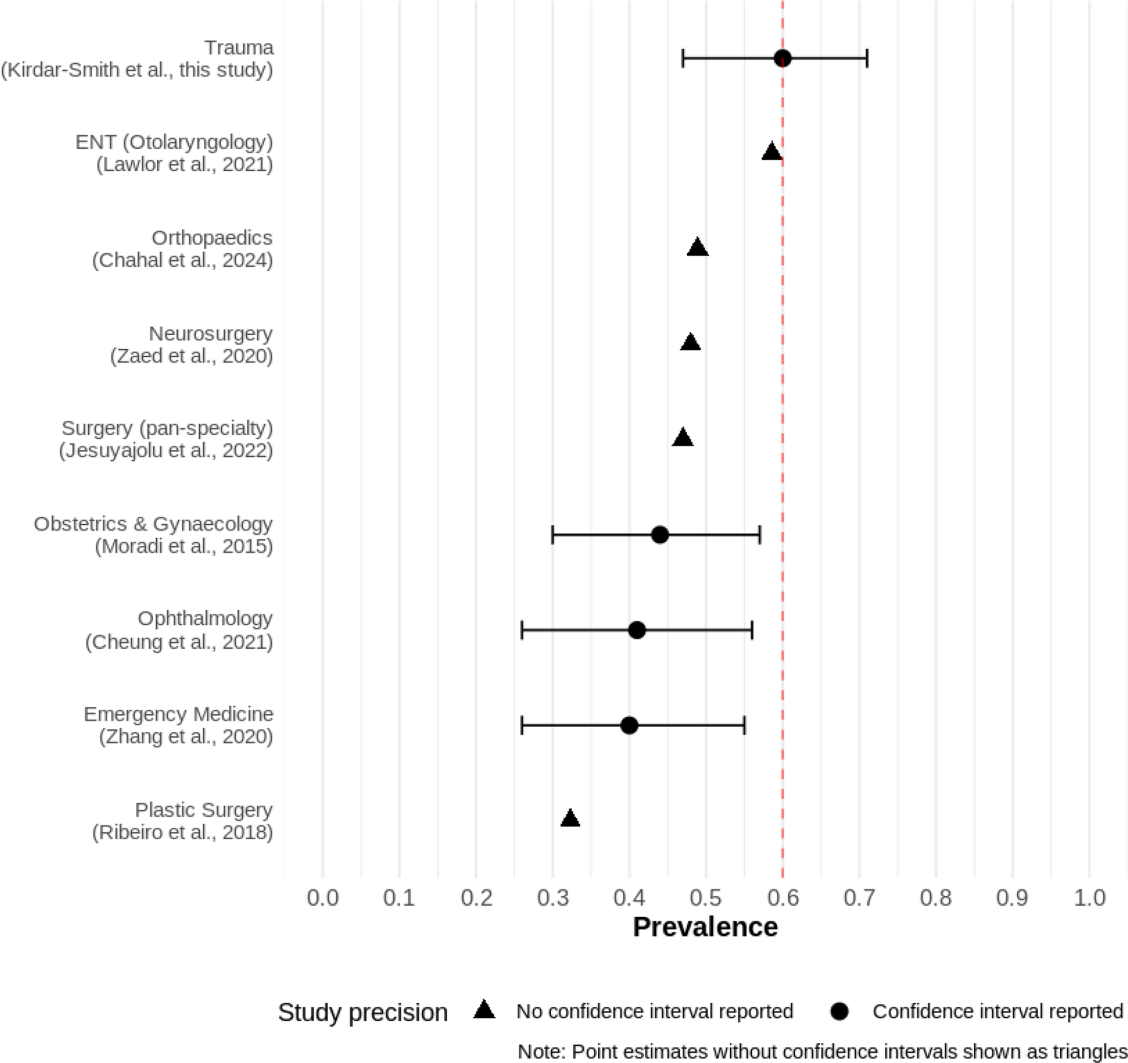
Forest plot comparison of this article’s burnout prevalence statistic versus other specialties.

Figure 3 gives a representation of the average burnout prevalence of trauma surgeons that was found in this article compared with average burnout statistics found by other meta-analyses conducted in other (majority surgical) specialties. The choice of studies included in figure 3 comprises predominantly surgical specialties; for which systematic reviews exist. The specialty of emergency medicine was also included as it is consistently found to be at high risk of burnout.^16 23^

**Figure 4:**
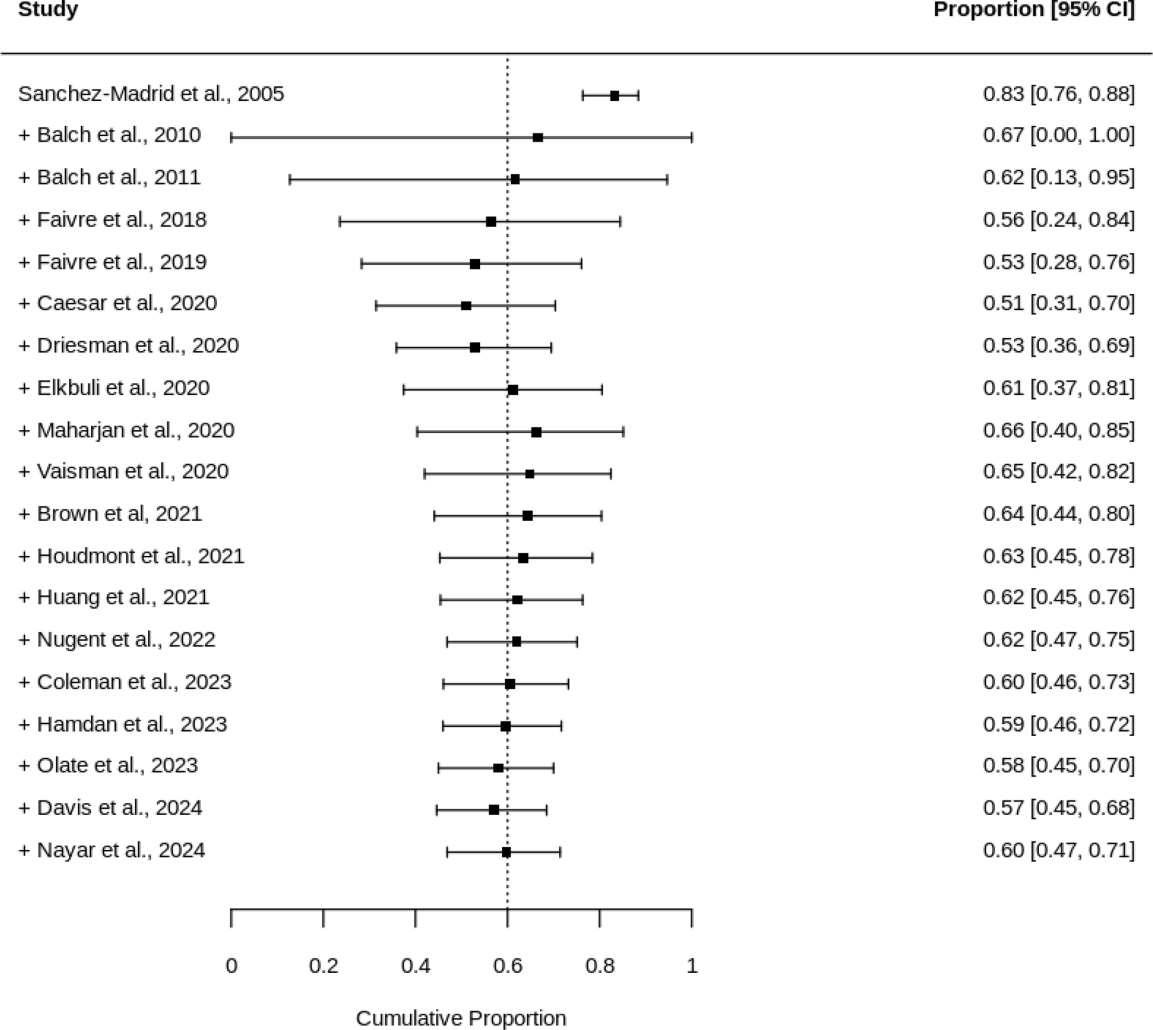
Cumulative proportion forest plot of burnout prevalence among trauma surgeons.

Study weights were relatively balanced, ranging from 2.8% to 5.7%, suggesting no single study dominated the analysis. Cumulative meta-analysis demonstrated initial instability in effect estimates, with convergence occurring after 2020, settling around 60–65% (figure 4). Evidence of publication bias was detected (Egger’s test p=0.0468), though trim-and-fill analysis did not suggest missing studies, as the adjusted estimate remained unchanged at 60.0% (95% CI 46.9% to 71.4%). The funnel plot (online supplemental figure 2) showed some asymmetry, with studies dispersed beyond the expected confidence limits.

Meta-regression comparing MBI and non-MBI studies showed no statistically significant difference (F(1,17) = 1.37, p=0.2583) between groups. Pooled estimates were 54.9% (95% CI 39.4% to 69.5%) for MBI studies (n=13) versus 69.1% (95% CI 47.5% to 84.7%) for non-MBI studies (n=6). The meta-regression model explained only 6.33% of the observed heterogeneity (R²=6.33%), with significant residual heterogeneity remaining (QE=326.42, p<0.0001). High heterogeneity persisted within both subgroups (MBI studies: I²=95.8%; non-MBI studies: I²=98.1%).

Trauma surgery is known for being a high-stakes specialty involving tasks in a cognitively and physically demanding environment.^32^ During meta-regression analysis comparing burnout between MBI and non-MBI studies (using the following auxiliary metrics: Copenhagen Burnout Inventory, Oldenburg Burnout Inventory, multiple choice questions, Likert-style questions, and non-specified surveys), the criteria for statistical significance was not met (p=0.2583). The minimal heterogeneity explained by measurement tool choice (R²=6.33%) suggests factors other than choice of burnout metric are more influential in determining burnout prevalence.

## Discussion

### Main findings

From our analyses, we identified the main burnout-exacerbating variables as: younger age^24^, hours worked^23^, poor sleep quantity or quality^31^, female gender^28^, working in the public sector^18^, and poor working conditions^16^. The key burnout-mitigating factors identified were: presence of family^26^, good work-life balance^22^, personal hobbies^30^, physical exercise^23^, permanent job contracts^18^, teaching or university hospitals^28^, mentorship^33^, and protected non-clinical time^34^.

One of the most identified burnout-exacerbating variables was younger age and relative inexperience.^9 14 16 17 23 24 31^ Many studies have found age to be a statistically significant factor associated with burnout,^24^ with studies demonstrating that younger trauma surgeons have a higher prevalence of burnout across all MBI components.^14^ A plausible reason for this inverse relationship between age and burnout could be explained by older surgeons having more time and experience to develop effective coping mechanisms. Also, regarding the variable of age, the survivorship effect must be acknowledged, in which some of the most burnt-out surgeons will quit and therefore not survive to be assessed by studies as older surgeons. It is commonplace for younger surgeons to work longer and more unsociable hours compared with older colleagues^17^. Trauma surgery regularly has the highest workload among specialties, with one study finding an average of 72.8 hours worked per week for trauma surgeons.^9^ Interestingly, in some cases, high working hours were not found to be associated with burnout, and they are rarely direct risk factors.^22^ This may be explained by trauma surgeons’ passion for their craft, reflected by consistently high levels of PA.^29^

It has long been theorized that burnout occurs more frequently in women,^35^ with studies commonly finding the same positive correlation between female gender and increased burnout rates.^25^ compared with age-matched male physicians, female physicians are more likely to be the primary childcare provider, and are consequently more likely to assume greater parental responsibility and maintain similar clinical duties to male counterparts.^36^ One study quantified this to constitute an additional 8.5 hours per week.^37^ Societal expectations of women, particularly in relation to childcare and domestic roles,^36^ feed into our proposed paradigm of a higher burnout prevalence among female trauma surgeons. This article found that female surgeons are more burnt out in the EE domain, whereas male surgeons are more burnt out in the DP domain,^38^ aligning with existing studies.^39^ This gender disparity across burnout domains can be attributed to a difference in coping strategies and patient interactions, as female physicians tend to engage in more psychosocial questioning,^40^ leading to greater occupational emotional demands.^19^ Male physicians tend to adopt ego-defense strategies,^19^ paralleling the phenomenon of DP. This gender disparity is only exacerbated by low female representation in trauma surgery. Most of the reviewed studies recorded a minority of female respondents, with a low of 6.3%.^21^ However, this is a systemic finding, with a recent study finding trauma and orthopedic surgery to have the lowest female representation (7.3%) of all surgical specialties.^41^ Despite this disparity, in-house call, a core part of acute care and trauma surgery, affects both sexes equally, further pointing to the fact that trauma itself, as a specialty, is a direct occupational risk factor for burnout.^42^

Sleep deprivation plays an immense role in the cause of burnout, with the link to overall burnout well substantiated.^43^ We found sleep duration and consistency to be predictors of daily burnout.^15^ The clinical ramifications include burnout^44^ and consequently, medical errors^43^. Extended periods of wakefulness and inconsistent sleep-wake times are inherent in trauma surgery and, as such, sleep-related impairment is an occupational risk of a career in such a specialty. Another factor linked to burnout is the working environment. From our analysis, we identified the main constituent variables to be: insufficient staff numbers^25^, impaired work relationships^16^, mental strain^45^, high-pressure environments^46^, and administrative stress^30^—electronic medical record (EMR) stress^47^ in particular, to the extent that studies postulate the primary root cause of physician burnout is the EMR itself^6^. This operational variable reinforces an established proposed typology of burnout risk factors into “operational” and “organisational” categorization,^48^ further cementing the criticality of the role work environment plays in influencing burnout.

Surgeons face excessive administrative tasks, on average spending 15.6% of total working time on such tasks.^49^ This increased administrative work is a key influential factor contributing to physician distress,^50^ as it is accepted that reducing administrative burden can mitigate burnout.^34^ One of the strongest burnout-mitigating factors identified in the literature was a good work-life balance;^22^ found to be protective of burnout in every mention. Specifically, trauma surgeons with children at home experienced higher levels of PA and lower incidences of burnout. Physicians have lower rates of satisfaction with work-life balance compared with the general population.^51^ However, among physicians, trauma surgeons have been reported to have the lowest mental quality of life and, resultantly, the highest rates of burnout of all surgical specialties.^9^

A less-researched factor regarding burnout is protected non-clinical (academic) time.^34^ Academic time involves allocated time consisting of no patient contact, instead providing opportunities to catch up on work or invest in academic interests without conflicting obligations.^52^ This time is associated with decreased EE, and increased surgeon well-being, supporting individual and institutional financial grants,^53^ without affecting surgical caseload or coverage.^34^ Physical exercise is another preventive factor,^31^ alongside hobbies outside of work.^30^ The presence of a ‘life outside work’ has an alleviating influence on burnout rates. Trauma surgery rotations leave less opportunity for burnout mitigation. On average, surgeons on trauma rotation exercised less than half as much time as those on non-trauma surgical rotations.^20^

Arguably, the most serious ramification of burnout is medical errors. Burnt-out surgeons are twice as likely to be involved in patient safety issues.^33^ To quantify this, a one-point increase in EE and DP constitutes an increase in the risk of major medical errors by 5% and 11%, respectively.^4^

### Strengths and limitations

Some of the main strengths of our study lie in our data collection and analyses. The predominance of the MBI is beneficial, facilitating intra-study comparison and statistical analysis. Another strength is the large study sample sizes, combined with our choice to dual-weight burnout prevalence statistics by sample size and risk of bias, as well as conducting univariate sensitivity analyses. This study is subject to a few limitations. First, as there are no universally accepted diagnostic cut-off scores for burnout, analyses were constrained by persisting academic incongruency as to what comprises a state of burnout. Issues inherent with collecting data on burnout are the recency effect and survivorship bias. Additionally, selection bias could potentiate inaccuracies in data collection. The design of most studies was cross-sectional—precluding analysis of cause and effect between identified variables.

### Implications, applications and future research needs

We calculated trauma surgeons to have the highest prevalence of burnout at 60.0%. This finding marks the most significant burnout prevalence statistic reported by meta-analyses that exist for surgical specialties (figure 3). Our study reinforces the existing paradigm within research that trauma surgeons regularly have the highest levels of burnout and a correspondingly low quality of life.^54^ Arguably, the most significant future repercussion of burnout among trauma surgeons is the perception of it. This serves as a primary deterrent to attracting medical students,^55^ leading to a mounting decline in interest among top medical students in pursuing surgical training.^56^

Existing data point to the fact that the specialty of trauma surgery is itself a risk factor for and directly associated with burnout^9^. However, despite its highly demanding nature, trauma surgery is a highly rewarding specialty.^57^ One feasible explanation is that in trauma surgery, there is simply a significant proportion of surgeons who can work under intense stressors. Nevertheless, they remain highly motivated, with high levels of PA fundamentally attributable to a love for their craft.

In this way, from both an academic and clinical perspective, it is prudent that we move away from a focus on diagnosing burnout, and instead target our finite resources at preventing and managing burnout and its consequences.

From our analyses, there are various evidence-based interventions that hold power to mitigate the effects and phenomenon of burnout itself. The responsibility for interventions and more broadly, physicians’ well-being must not fall on the already burdened shoulders of physicians themselves, but instead on the shoulders of organizations and governing bodies. We recommend bottom-up, peer-facilitated programs allowing for flexible interventions, without exacerbating and perpetuating burnout itself. First proposed in 2003, the Trauma Risk Management model was introduced for use in the UK Armed Forces as a post-traumatic management strategy based on peer-group risk assessment,^58^ providing baseline support and identifying individuals in need of additional support.^59^ Another similar intervention proven to mitigate burnout among surgeons is rotational debriefing. As aforementioned, it is known that trauma surgeons experience high-stakes and often traumatic cases on a regular basis. Structured team debriefings at regular intervals, or after critical or mass casualty incidents, facilitate emotional processing, mental health dialogue, and can be built into existing morbidity and mortality conferences—an infrastructure already in place at many trauma centers. Additionally, the adoption of a formal mentorship scheme, with an ‘opt-out’ design increases physicians’ comfort, and is associated with high utilization rates.^60^ Mentorship is accepted as a burnout-mitigating variable,^33^ and the importance of good leadership in a clinical setting has a direct effect on the personal well-being of mentored surgeons.^61^ The introduction of protected non-clinical time is an established protective variable.^34^

However, the stigma around mental health, especially in the medical field, persists and hinders both the reporting and management of burnout.^62^ Views that mental health diagnoses are a sign of weakness are endemic in the medical profession, accompanied by perceptions of shame and embarrassment,^63^ leading to reduced help-seeking behaviors.^62^ It is foundationally critical to both tackle the stigma surrounding mental health and to ensure organizational responsibility for burnout before any significant change can occur.

## Conclusion

This systematic review and meta-analysis provides evidence that trauma surgeons experience burnout at one of the highest rates of all medical specialties. As well as more frequent and intense pan-specialty generic risk factors, unique risk factors for burnout inherent to the specialty of trauma surgery exist. These findings reinforce the necessity to intervene in an evidence-based manner, putting the surgeon first, and accepting, on an organizational level, that burnout is an occupational phenomenon. Our analyses and data point to the finding that trauma surgery itself is an academically and clinically validated risk factor for burnout. However, the fact remains that in the face of innumerable profound occupational stressors, trauma surgeons maintain consistently high levels of PA and individual fulfillment.

## Supplementary material

10.1136/tsaco-2025-001873 online supplemental file 1

## Data Availability

All data used within this meta-analysis and R code are available here: https://github.com/ricardotwumasi/burnout-trauma-surgeon.
