# Supplementary material for: Burnout among trauma surgeons: a systematic review and meta-analysis
Source: Trauma Surg Acute Care Open. 2025 Oct 28;10(4):e001873. doi: 10.1136/tsaco-2025-001873 (PMC12570958; doi:10.1136/tsaco-2025-001873)
Supplement: online supplemental file 1 [file tsaco-10-4-s001.pdf]

SUPPLEMENTARY MATERIALS

Reporting Criteria	Reported in Study	Reported on Page No. / Fig.
**Reporting of Background**		
Problem definition	Yes	2
Hypothesis statement	Yes	2
Description of study outcome(s)	Yes	3
Type of exposure/intervention used	Yes	3
Type of study designs used	Yes	3
Study population	Yes	2
**Reporting of Search Strategy**		
Qualifications of searchers (e.g. librarians & investigators)	Yes	3
Search strategy, including time period included in synthesis and keywords	Yes	3
Effort to include all available studies, including contact with authors	Yes	3
Databases and registries searched	Yes	3
Search software used, name and version, including special features used (e.g. explosion)	Yes	4
Use of hand searching (e.g. reference lists of obtained articles)	Yes	3
List of citations located and those excluded, including justification	Yes	3
Method of addressing articles published in languages other than English	Yes	3
Method of handling abstracts and unpublished studies	Yes	3
Description of any contact with authors	Yes	4
**Reporting of Methods**		
Description of relevance or appropriateness of studies assembled for assessing the hypothesis to be tested	Yes	3
Rationale for the selection and coding of data (e.g. sound clinical principles or convenience)	Yes	3
Documentation of how data were classified and coded (e.g. multiplier raters, blinding and interrater reliability)	Yes	3
Assessment of confounding (e.g. comparability of cases and Controls in studies where appropriate)	N/A – prevalence study with no controls	N/a
Assessment of study quality, including blinding of quality assessors; stratification or regression on possible predictors of study results	Yes	3
Assessment of heterogeneity	Yes	4
Description of statistical methods (e.g. complete description of fixed/random effects etc.) in sufficient detail to be replicated	Yes	4
Provision of appropriate tables and graphics	Yes	4-6
**Reporting of Results**		
Graphic summarising individual study estimates and overall estimate	Yes	Fig. 2
Table giving descriptive information for each study recorded	Yes	Table 1

Results of sensitivity testing (e.g. subgroup analysis)	Yes	Table 2
Indication of statistical uncertainty of findings	Yes	5
**Reporting of Discussion**		
Quantitative assessment of bias (e.g. publication bias)	Yes	SM Fig. 2
Justification for exclusion (e.g. exclusion of non-English language citations)	Yes	3
Assessment of quality of included studies	Yes	3
**Reporting of Conclusions**		
Consideration of alternative explanations for observed results	Yes	6-8
Generalisation of the conclusions (e.g. appropriate for the data presented and within the domain of the literature review)	Yes	9
Guidelines for future research	Yes	9
Disclosure of funding source	Yes	9

SM Figure 1: MOOSE Checklist

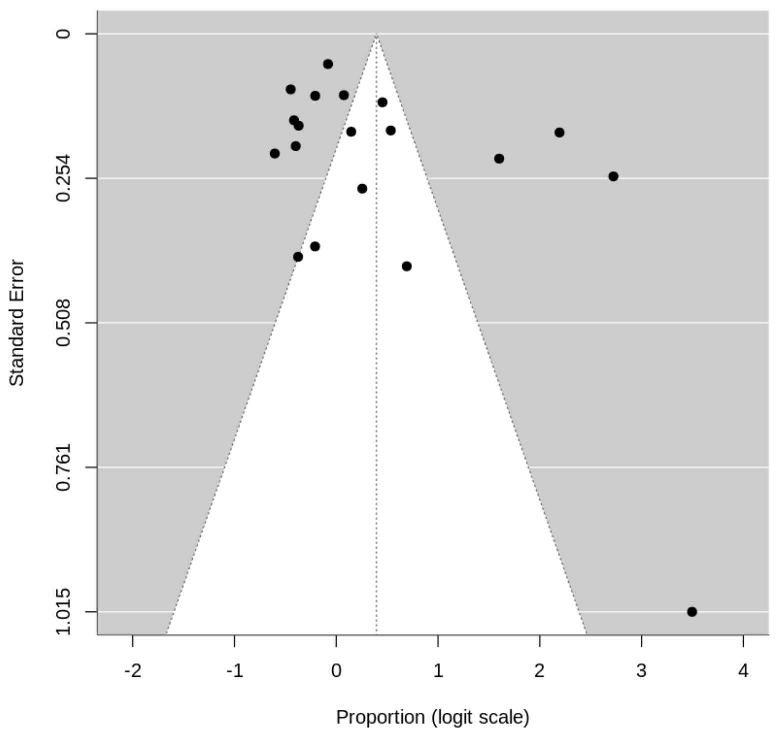

SM Figure 2: Funnel Plot of Standard Error by Logit Proportion
